# The Identification and Challenges of Dopamine Dysregulation Syndrome: A Case Report

**DOI:** 10.7759/cureus.86547

**Published:** 2025-06-22

**Authors:** Andrew Sulaiman, Idris Leppla

**Affiliations:** 1 Psychiatry, Johns Hopkins University, Baltimore, USA

**Keywords:** case presentation, cl psychiatry, dds, dopamine dysregulation syndrome, parkinson' s disease

## Abstract

A 48-year-old man with a history of opioid use disorder (unmanaged) and presumed Parkinson's disease presented to the emergency department secondary to worsening of his Parkinson's tremors/ambulatory dysfunction. He expressed that he was taking up to 20 doses of his carbidopa/levodopa (25mg-250mg pills for a total of >5g levodopa every day). The patient was admitted for the management of Parkinson’s tremors and substance abuse. He was administered carbidopa/levodopa (25mg-250mg) every eight hours. Psychiatry was consulted due to marked functional fluctuations in his presentation and found that he demonstrated positive choreiform movements of the head/neck and upper extremity after taking the carbidopa. The patient's presentation prompted a suspicion of dopamine dysregulation syndrome (DDS). Supporting evidence included that the patient was insistent that the current regimen of carbidopa/levodopa wasn’t managing symptoms, and even when the frequency was increased to every six hours, the patient expressed that his symptoms were still present and problematic. The patient stated that if the carbidopa/levodopa dosage wasn’t further increased, he would leave the hospital. He ended up leaving against medical advice and was later ultimately re-admitted with the goal of transitioning to a rehabilitation facility for the treatment optimization of his substance use disorder and Parkinson's disease.

This case demonstrates the challenges and presentation of DDS. DDS is a disorder marked by compulsive overuse of dopaminergic medication, which frequently occurs in individuals with Parkinson's disease. Additionally, this case reviews diagnostic criteria and moreover summarizes literature supporting methods of clinical management.

## Introduction

Parkinson's disease is characterized by degradation of the substantia nigra leading to diminished levels of dopamine. As such, dopamine replenishment via medications such as levodopa is a mainstay for the treatment of Parkinson's disease. However, evidence has shown that this dopamine agonist may carry addictive properties when utilized in Parkinsonian patients, leading to disinhibited behavior [1,2]. Careful evaluation of patients with increased usage of levodopa is important, as this may be an indicator of dopamine dysregulation syndrome (DDS), which is characterized by seven main criteria. The criteria include compulsive use of dopaminergic medications at high concentrations (exceeding what is typically used for Parkinson’s disease management) for at least six months in duration, impairment of social and/or occupational functioning, demonstration of what appears to be drug seeking behavior regarding dopaminergic medication, a stark opposition against reducing dopaminergic medication dosage and apparent withdrawal symptoms once dopaminergic medication is reduced [3].

The precise prevalence of DDS in patients with Parkinson's disease is yet to be determined; however, multiple cohorts have shown a DDS frequency of ~3% in patients with Parkinson’s disease [3]. These cases are rare, diagnostically challenging, and easy to miss in a differential diagnosis. As such, early recognition is important to prevent substance dependence on dopaminergic medications and its associated features of dyskinesia, mood/behavior changes, hypomanic features, psychological tolerance, and aggression [4]. We present a complex presentation of DDS in a man with a presumptive but unconfirmed diagnosis of Parkinson's disease for which the psychiatry service was consulted and use this case to demonstrate the challenges of DDS, review diagnostic criteria, and moreover summarize the literature supporting methods of clinical management.

## Case presentation

The patient is a 48-year-old man with a history of depression, gastroesophageal reflux disease, hepatitis C (treated), opioid use disorder (unmanaged and ongoing for the past 23 years), and Parkinson's disease who presented to the emergency department secondary to worsening of his Parkinson's tremors/ambulatory dysfunction. The patient dropped out of school in the 8th grade, was not recently employed but worked in construction prior to being on disability, which he attributed to his Parkinson's disease. He was undomiciled at presentation, his marital status was single, he denies any trauma/abuse, and he denies other drug use aside from heroin/fentanyl. He uses heroin/fentanyl through multiple routes (intranasally and intravenously) and describes the frequency of his usage as daily. He has a history of suboxone treatment (buprenorphine-naloxone 2 mg-0.5 mg) but discontinued this medication last year after 5-6 years of treatment as he didn't like the idea of being dependent on medication. He then maintained sobriety for 5-6 months with his relapse occurring a few months prior to presentation. There was no family history of movement disorders or Parkinson's disease, and per collateral, there was no family history of opioid use disorder.

He was only on one medication at presentation, carbidopa/levodopa, which he stated that he takes up to 20 doses of 25 mg-250 mg pills for a total of >5g levodopa daily. For context, the typical maximum daily dose of carbidopa is 200 mg/day and levodopa is 2 g/day; it was unclear when he first started taking such high doses of carbidopa/levodopa, but he was prescribed the medication after getting diagnosed with Parkinson's disease five years prior to presentation. He stated that carbidopa/levodopa helped control motor issues and improve his functionality, but he also confirmed a correlation between increased carbidopa/levodopa usage and an impairment in social/occupational functioning (lost his job, lost his house, and he has no friends or family support). The patient's reporting of his prescribed dosing of carbidopa/levodopa and his source of this medication could not be verified. It seemed to be obtained from a combination of emergency department visits and providers across multiple states. A standard urine immunoassay was positive only for fentanyl (reported usage two days prior to the admission), but the patient’s initial presentation was not suspicious of amphetamine, opioid, or benzodiazepine withdrawal (Clinical Opiate Withdrawal Scale score was negative during admission). Upon review, it was found that the patient didn’t have a formal diagnosis of Parkinson’s and was placed on carbidopa/levodopa by a primary care provider 4-5 years ago for tremors, from which his dosage has been getting progressively greater.

The patient was admitted to the inpatient medicine unit for the management of Parkinson’s tremors and substance abuse management with the goal of discharging to a substance abuse treatment program/inpatient rehabilitation placement. He was placed on a clinical opiate withdrawal scale protocol and a Subutex treatment protocol. Due to his history and pill-rolling tremor, he was also administered carbidopa-levodopa 25 mg-250 mg three times a day. Neurology was consulted and he was given a working diagnosis of young idiopathic Parkinson’s disease and was administered carbidopa/levodopa (25 mg-250mg) every eight hours. Psychiatry was consulted due to marked functional fluctuations and it was during the psychiatry work-up that the diagnosis of DDS was put in the differential diagnosis.

Extensive workup including chest X-rays and laboratory investigations was unrevealing with the exception of a mildly elevated creatine kinase indicating mild rhabdomyolysis (treated and resolved during his admission). Vitals were within normal limits and a physical exam revealed a low-amplitude resting tremor in the bilateral hands and left foot/ankle (distractible). A simple upper extremity kinetic tremor was also demonstrable during interviews which again fluctuated in severity and was distractible in nature. Patient demonstrated intermittent festinating gait and intermittent mild scissoring gait particularly when facing obstacles other distractions with fluctuating performance on activities of daily living and distance examinations. Some days he was able to walk 300 feet and other days he was unable to walk at all; these fluctuations were without a clear pattern related to carbidopa/levodopa dosing.

A mental status examination was performed, which revealed a disheveled and poorly groomed individual who was alert, calm, and cooperative. While no psychomotor agitation or retardation was noted, there were abnormal right-hand tremors for brief moments during the exam. Speech was normal in volume, tone, and rhythm and no formal thought disorder was elicited. Mood was slightly constricted with some affective brightening, the patient's self-attitude was normal and there was no passive death wish or suicidal ideation demonstrated. Insight and judgement were fair, and he was oriented to person, place, time, and general circumstances. The exam was negative for signs of psychosis, depression, and delirium. There were signs of hypomania, but these symptoms occurred in the context of substance abuse, which confounded the assessment (the patient would state that he would feel invincible and be awake for three days straight). Montreal Cognitive Assessment (MOCA) provided a score of 26/30 with deficits in delayed recall. The patient denied punding, pathological gambling, hypersexuality, and compulsive shopping or compulsive eating. No other psychometric testing was performed.

The patient expressed that his carbidopa/levodopa regimen (every eight hours) was inadequate for symptom management, and the medication was only effective for two hours after which he was restricted in movement and bedbound. He also demonstrated positive choreiform movements of the head/neck and upper extremity after taking the carbidopa/levodopa and expressed that he experienced visual hallucinations when on higher dosages. He was adamant that the dosage/frequency of carbidopa/levodopa was inadequate and due to nighttime exacerbation, medication administration was increased to every six hours on day 12 of admission. There was marked resistance anytime carbidopa/levodopa dose reduction was discussed, and the patient was hyper-focused (clock-watching) regarding the next administration of medicine. The increase in medication frequency still couldn’t effectively manage the patient, and he expressed that unless his dosage was increased, he would leave the hospital against medical advice (AMA). Despite care coordination being performed to manage this patient’s complex psychosocial presentation and substance use disorder via psychotherapy, coping strategy development and placement to an inpatient substance abuse treatment facility, he left the hospital AMA. He later returned to the emergency department overnight after using intranasal heroin and requested medications for opioid use disorder initiation. He was admitted again to the internal medicine unit and after a five-day stay, he was transitioned to a skilled nursing facility for buprenorphine titration and substance use disorder management as well as for Parkinson's treatment. 

## Discussion

This patient demonstrated Parkinsonian features with marked resolution upon carbidopa/levodopa administration for a limited timeframe (two hours). In assessing any medication interactions that could lead to reduced carbidopa/levodopa levels, dopamine D2 receptor antagonists and iron salts are thought to decrease the therapeutic effect of levodopa, but the patient was on none of these medications [5].

There was marked resistance anytime carbidopa/levodopa dose reduction was discussed, and the patient was hyper-focused (clock-watching) regarding the next administration of medicine. Based on his presentation and his impairment in social/occupational functioning (lost his job and house, no friends or close family he met criteria for DDS, aka hedonistic homeostatic dysregulation) (criteria described in Table 1) [3]. Functional neurological disorder was on the differential diagnosis and while there were overlaps in clinical symptoms (altered motor function, cause of significant distress and impairment in daily life) as the patient’s condition could be better explained due to DDS, per the Diagnostic and Statistical Manual of Mental Disorders-V (DSM-V), functional neurological disorder was placed lower on the differential diagnosis [6].

**Table 1:** Diagnostic Criteria for Dopamine Dysregulation Syndrome DDS: Dopamine dysregulation syndrome

	Diagnostic Criteria for Dopamine Dysregulation Syndrome***
1	A presumptive diagnosis of Parkinson's Disease (a diagnosis which can be made clinically)
2	The time frame is >6 months in duration.
3	Increased dosages of Dopaminergic medication, greater than what is typically seen to alleviate Parkinsonian symptoms (e.g.,>2g per day of levodopa).
4	Desire for additional dopaminergic medication dosage/frequency to manage Parkinsonian symptoms (appearing similar to drug-seeking behavior), possible elaboration/atypical Parkinsonian symptoms, dyskinesis/choreiform movement when on Dopaminergic medication, hoarding of medication, strong opposition when Dopaminergic medication is reduced.
5	The presence of cyclothymic affective syndrome, hypomanic or manic episodes, when taking dopaminergic medication.
6	Once Dopaminergic medication is reduced, the development of withdrawal symptoms, which include depression, irritability, anxiety, or dysphoria.
7	Dopaminergic Dysregulation Syndrome can lead to loss of social (issues with family and friends) and occupational functioning (job), legal difficulties, poor coping methods, and possible violent behaviors.
***	These seven criteria above are adopted from the study by Giovanni et al. (2000) [3] and act as provisional criteria to aid in the recognition of DDS.

It is hypothesized that the mechanism underlying DDS may reside in dopaminergic sensitization of the mesolimbic system [7]. Management of DDS is complex, as there are no set guidelines, but recommendations based on the literature, which we have summarized below.

There should be a consideration for DDS in patients with Parkinsonian features who are also taking >2000 mg daily of Levodopa. However, it is important to note that DDS is not uniquely identified in Parkinson's patients; DDS has been diagnosed in patients with fibromyalgia, advanced uremic neuropathy, restless legs syndrome, and macroprolactinoma [8]. It has been thought that DDS could theoretically arise in any population at risk for dopamine medication abuse. Symptoms to be aware of are novelty seeking behavior and impulsivity, and to be aware that the main driver behind DDS is the need to overcome an incapacitating motor dysfunction through increasing medication despite symptoms of toxicity and withdrawal [8].

Once patients with DDS are admitted for treatment, levodopa dosage and frequency should follow a strict regimen and optimization utilizing agents including catechol-O-methyltransferase inhibitors, tolcapone, and/or monoamine oxidase B medications with recommendations provided by neurology [9]. While the patient may state that the prescribed regimen is inadequate in symptom management, rescue doses of carbidopa/levodopa should be avoided/rationed [3]. Throughout their admission, the patient should be monitored to ensure no covert medicine administration.

There should also be a formal psychiatric assessment performed, and additionally, a cognitive assessment may be of benefit, especially in cases where substance use disorders are involved, to determine any influence on executive function. It has been found that in DDS patients, acute psychosis can be controlled with olanzapine, clozapine, quetiapine, or risperidone [9]. Delusions, psychosis, and aggressive behaviors are managed with the same antipsychotics, and it was found that clozapine was more effective than quetiapine in managing these symptoms [9]. For depression, selective serotonin reuptake inhibitors/serotonin and norepinephrine reuptake inhibitors can be prescribed; however, these will not affect the addictive behaviors of the patients [9]. In one patient, memantine was associated with cessation of levodopa cravings. Other reports have highlighted the utility of sodium valproate, which effectively treated five cases of DDS [10]. Additionally, other medications such as continuous infusions of apomorphine and Duodopa have shown efficacy, and there is support for the utility of deep brain stimulation to help control impulsivity and compulsivity symptoms [11]. While promising, further trials are required for definitive proof [10,12]. Upon discharge, the patient should be given their medication under the supervision of caregivers or family, if feasible, to ensure compliance. This factor is actually the most important non-pharmacological factor associated with remission of DDS [9]. Finally, if the patient is agreeable, enrollment in a drug addiction rehabilitation program would also be beneficial.

## Conclusions

Here, we report a case of DDS and highlight a typical presentation. Unfortunately, in our case the patient left AMA before further treatment could have been discussed. However, we highlight recent literature and provide a framework on the management of DDS for cases amenable to medical intervention. Further research/clinical trials into diagnostic methods/criteria for DDS and treatment regimen will be of direct clinical benefit. It is easy for DDS to not appear on a differential diagnosis or to be mistaken for disease progression or psychiatric comorbidity. As such, education of DDS to both patients and providers would also be of tremendous importance so that DDS may be identified early to prevent psychosocial distress and ensure that multidisciplinary, evidence-backed treatment can be initiated for the benefit of the patient.
